# Superior Technique for the Production of Agarose Dressing Containing Sericin and Its Wound Healing Property

**DOI:** 10.3390/polym13193370

**Published:** 2021-09-30

**Authors:** Supamas Napavichayanun, Prompong Pienpinijtham, Narendra Reddy, Pornanong Aramwit

**Affiliations:** 1 Department of Pharmacy Practice, Faculty of Pharmaceutical Sciences and Center of Excellence in Bioactive Resources for Innovative Clinical Applications, Chulalongkorn University, Bangkok 10330, Thailand; snupamas@gmail.com or; 2 Department of Chemistry, Faculty of Science, Chulalongkorn University, 254 PhayaThai Road, Phatumwan, Bangkok 10330, Thailand; Prompong.P@chula.ac.th; 3 Jyothy Institute of Technology, Karnataka 560082, India; narendra.r@ciirc.jyothyit.ac.in; 4 The Academy of Science, The Royal Society of Thailand, Dusit, Bangkok 10330, Thailand

**Keywords:** agarose, sericin, glycerin, scaffold, freeze-thawing, freeze-drying

## Abstract

Finding a simple and eco-friendly production technique that matches to the natural agent and results in a truly valuable natural scaffold production is still limited amongst the intensively competitive natural scaffold development. Therefore, the purpose of this study was to develop natural scaffolds that were environmentally friendly, low cost, and easily produced, using natural agents and a physical crosslinking technique. These scaffolds were prepared from agarose and sericin using the freeze-drying method (D) or freeze-thawing together with the freeze-drying method (TD). Moreover, plasticizers were added into the scaffold to improve their properties. Their physical, mechanical, and biological properties were investigated. The results showed that scaffolds that were prepared using the TD method had stronger bonding between sericin and other compounds, leading to a low swelling ratio and low protein release of the scaffolds. This property may be applied in the development of further material as a controlled drug release scaffold. Adding plasticizers, especially glycerin, into the scaffolds significantly increased elongation properties, leading to an increase in elasticity of the scaffold. Moreover, all scaffolds could activate cell migration, which had an advantage on wound healing acceleration. Accordingly, this study was successful in developing natural scaffolds using natural agents and simple and green crosslinking methods.

## 1. Introduction

The wound healing process is composed of four major phases, which are the coagulation and hemostasis phase, the inflammatory phase, the proliferation phase, and the remodeling phase. Coagulation and hemostasis are the first steps to occur after skin injury [1]. Then, the inflammatory cells and mediators are triggered to the wound area, causing swelling, heat, and redness [2]. In the proliferation phase, the epithelial cells are migrated, and collagen is regenerated to cover the wound [3]. Lastly, the collagen fibre, tissue, and matrix are rebalanced in the remodeling phase [4]. All new tissue formation processes begin between two and ten days after skin injury [5]. However, an underlying disease, poor nutrition, infection, and inappropriate wound management lead to delayed wound healing, which may progress to a chronic wound and organ loss [6]. A suitable covering, which protects wound infection and provides an appropriate wound environment, is one of the important factors for the reduction of delayed wound healing [7]. Many wound dressings are developed to provide a competitive advantage during wound healing, such as providing protection against infection, keeping the wound moist, elimination of exudate, wound healing acceleration, mechanical protection, easy removal, biocompatibility, biodegradation, and non-toxicity [8,9,10,11,12]. The number of chronic wounds has risen following an increase in the elderly and diabetic population [13]. Therefore, wound dressing requirements also increase continuously. However, the increase in medical production results in a high amount of pollution and waste [14,15,16,17]. Green technologies, which are an environmentally friendly process, play an important role in solving these problems [18]. They are a highly competitive area of pharmaceutical research and production development [19]. For wound dressing development, finding a technique that is clean, safe, and cost-effective to create a new dressing while maintaining the basic dressing properties is still challenging. Because of the increase in wound dressing requirements, the successful research and development of an eco-friendly wound dressing produced by green methods may reduce a large amount of waste that is produced by conventional dressing production. Freeze-drying and freeze-thawing are physical crosslinking techniques that are used in biomaterial preparations [20,21,22,23,24,25]. Physical crosslinking is advantageous because it is safe and avoids toxic organic solvent or crosslinking agents, resulting in a harmless biomaterial product and reasonable cost [26]. Other crosslinking techniques are interesting processes in wound dressing production, such as supercritical carbon dioxide, which can use recycled carbon dioxide after processing and allows easy removal of the carbon dioxide from the final product, resulting in no chemical residue in the product [27]. However, a good quality, large sized supercritical carbon dioxide machine and good control of a complex system are intensively required [28]. Therefore, developing freeze-drying and freeze-thawing conditions that are simple and effective techniques for wound dressing production is strongly valuable.

For wound dressing materials, natural and synthetic polymers are commonly used as the dressing material, in the form of films, hydrogels, and scaffolds [29]. Recently, natural materials have gained much attention in biomedical production because of their high biocompatibility and environmentally friendly properties [30]. Polyhydroxyalkanoates (PHAs) are one of the new natural polymers that are synthesized by microorganisms using waste as a carbon source [31]. They have good mechanical properties and biocompatibility, leading to their use in wound dressing production [32]. However, there is a concern that their purity and chemical composition might induce an inflammatory reaction [33]. Other natural agents such as agarose, alginate, and carrageenan are polysaccharides that are also used in wound dressing production [34]. Because of the high porosity and non-adhesive properties of alginate dressing, it requires a secondary dressing to fix them [35]. Moreover, the alginate dressing was reported to be difficult to remove from the wound [36]. Carrageenan also have limitations in processability and reactivity, so modification of their chemical and physical properties, such as blending with other polymers, is required to improve their properties [37]. Agarose is a linear polymer that consists of β-1,3-linked d-galactopyranose and α-1,4-linked 3,6-anhydro-α-l galactopyranose [38]. It is generally extracted from seaweed. Because of its biocompatibility, transparency, and neutral charge, it is used in many biomaterial applications, including wound dressing and tissue engineering [39,40]. Moreover, an agarose scaffold has shown excellent mechanical properties and a suitable porous structure for drug delivery [41]. The agarose melting point is around 80–90 °C, while the agarose gelation point is 30–40 °C [42]. Therefore, thermal crosslinking methods, including the freeze-drying technique and the freeze-thawing technique, are used to prepare agarose hydrogel. Accordingly, agarose is suitable for use as a dressing material. Furthermore, adding a wound healing accelerating agent into the wound dressing could improve the performance of the wound dressing. It would make the wound dressing more valuable. Sericin, collagen, and epidermal growth factor are natural activating wound healing agents [43,44,45]. Sericin is a natural protein from the silk cocoon, which is a common waste product in the fabrication industry [46]. Nonetheless, it is a biocompatible material and has several biomedical properties, including antioxidant, anti-inflammation, and wound healing acceleration effects [47]. It can activate fibroblasts to promote collagen type I synthesis, leading to wound healing promotion [48]. Therefore, using sericin as a wound healing agent not only improves the wound dressing property but also reduces the amount of waste released into the environment. The brittleness of the dressing is also an important problem, so natural plasticizers were added into the dressing production in this study. Natural polyols, including glycerin and propylene glycol, are plasticizers that have low toxicity and biocompatibility [49]. They are hydroscopic molecules that enhance flexibility and prevent dressing brittleness [50]. Therefore, this study developed a natural wound dressing containing natural agents, using a non-toxic process. The physical, mechanical, and biological properties of the dressing including swelling properties, protein release profile, gel fraction analysis, structural analysis, elongation and Young’s modulus, cytotoxicity, and cell migration were intensively evaluated.

## 2. Materials and Methods

### 2.1. Materials

*Bombyx mori* cocoons were supplied by the Chul Thai Silk Co. Ltd., Phetchabun, Thailand. Sericin solution (SS) was prepared from the cocoons using a high-temperature and high-pressure degumming method [51]. Briefly, pieces of cocoon were autoclaved at 121 °C and 15 lbf/in^2^ for 60 min. The concentration of SS protein was measured using a BCA protein assay kit (Thermo Fisher Scientific, Waltham, MA, USA) at a wavelength of 562 nm. Bovine serum albumin (Thermo Fisher Scientific, Waltham, MA, USA) was used to prepare a standard curve for the protein calculation. Glycerin and propylene glycol were analytical grade from Ajax Finechem, New South Wales, Australia. Agarose and other chemicals were purchased from Sigma-Aldrich, Saint Louis, MO, USA.

### 2.2. Scaffold Preparation

**Figure 1:**
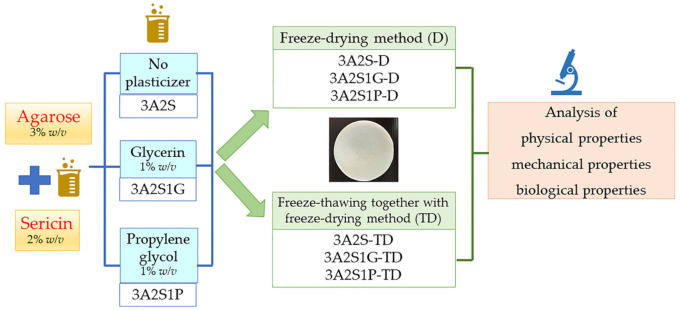
A graphical scheme of the study design.

Three formulations of agarose containing sericin scaffolds were investigated. A graphical scheme of the study design is shown in Figure 1. Agarose was completely dissolved in reverse osmosis water at 80 °C for 120 min. For the 3A2S scaffold, SS was added into the agarose solution to a final concentration of 3% *w/v* agarose and 2% *w/v* sericin. For the 3A2S1G scaffold, SS and glycerin were mixed into the agarose solution to a final concentration of 3% *w/v* agarose, 2% *w/v* sericin, and 1% *w/v* glycerin. For the 3A2S1P scaffold, SS and propylene glycol were added into the agarose solution to a final concentration of 3% *w/v* agarose, 2% *w/v* sericin, and 1% *w/v* propylene glycol. Each formulation was then poured into molds.

#### 2.2.1. Freeze-Drying Method (D)

The 3 different formulations (3A2S, 3A2S1G, 3A2S1P) were frozen at −20 °C for 72 h and were then dried using lyophilization (Heto LL 3000 lyophilizer, Allerod, Denmark) for 72 h (3A2S-D, 3A2S1G-D, 3A2S1P-D).

#### 2.2.2. Freeze-Thawing and Freeze-Drying Method (TD)

For the freeze-thawing method, each formulation (3A2S, 3A2S1G, 3A2S1P) was frozen at −20 °C for 16 h and then thawed at 25 °C for 8 h. This process was counted as 1 cycle. Each formulation was prepared using 8 cycles of the freeze-thawing method and then frozen at −20 °C for 72 h and dried using lyophilization (Heto LL 3000 lyophilizer, Allerod, Denmark) for 72 h (3A2S-TD, 3A2S1G-TD, 3A2S1P-TD).

For the swelling, protein release, and gel fraction tests, all scaffolds were cut into 1 × 1 cm^2^ pieces. Each formulation was tested in triplicate.

### 2.3. Analysis of Physical Properties

#### 2.3.1. Swelling Properties

Each scaffold was weighed before starting. It was then immersed in 5 mL of phosphate buffer saline (PBS, pH 7.4) at 37 °C. The swollen scaffold was carefully weighed at different periods, until its weight was unchanged, to evaluate the water content. The percentage of swelling was calculated using the following equation

Swelling (ratio) = (W_t_ − W_0_)/W_0_ (1)

where W_t_ was the swollen weight at different periods and W_0_ was the initial weight.

#### 2.3.2. Protein Release Profile

Each scaffold was immersed in 5 mL of PBS (pH 7.4) at 37 °C. At various time intervals, the PBS solution was collected and was shaken. The amount of released SS was measured using the BCA protein assay kit (Thermo Fisher Scientific, Waltham, MA, USA) at a wavelength of 562 nm. The concentration of released SS was calculated compared to the albumin standard curve.

#### 2.3.3. Gel Fraction Analysis

Each scaffold was weighed and then immersed in 5 mL of reverse osmosis water at 25 °C for 24 h. It was then incubated at 50 °C. The scaffold was weighed every 4 h until its weight was unchanged (dry weight). The percentage of gel fraction was calculated using the following equation

Gel fraction (%) = (W_d_/W_0_) × 100 (2)

where W_d_ was the unchanged weight after drying in the incubator and W_0_ was the initial weight.

#### 2.3.4. Structural Analysis Using Fourier-Transform Infrared Spectroscopy

All IR spectra were collected by a Thermo Scientific Nicolet iS5 Fourier-transformed infrared (FTIR) spectrometer (Thermo Fisher Scientific, Waltham, MA, USA) in an attenuated total reflection (ATR) mode using an iD7 ATR accessory and were recorded by a deuterated triglycine sulphate (DTGS) detector with a scan number of 16 and a spectral resolution of 4 cm^−1^. All spectra were presented without any modification, except normalization. The degree of crystallinity of agarose in the scaffold was determined by observing the packing of cellulose chains from CH_2_ deformation vibration in crystalline cellulose.

### 2.4. Analysis of Mechanical Properties

Each scaffold was cut into a dumbbell shape with the smallest dimension of 20 × 40 mm^2^. The thickness of each scaffold was exactly measured using Vernier calipers. Tensile testing was performed using a universal testing machine (Shimadzu EZ-S, Tokyo, Japan) with a load cell of 500 N and a speed of 5 mm/min. Each scaffold was investigated five times. The maximum stress, percentage of elongation, and Young’s modulus were calculated following stress–strain graph.

### 2.5. Analysis of Biological Properties

#### 2.5.1. Cytotoxic Assay

PrestoBlue^TM^ reagent ((Thermo Fisher Scientific, Waltham, MA, USA) was used to evaluate the influence of each scaffold on cell viability. Cells were seeded in 96 well plates at a density of 1 × 10^4^ human keratinocytes (HaCaT cells) per well and 5 × 10^3^ human dermal fibroblast (HDF) cells per well in cell culture medium (Dulbecco’s modified Eagle’s medium (DMEM)) and incubated for 24 h to allow cell adherence. Post incubation, cells were treated with different concentrations (12.5%, 25%, 50%, and 100%) of the released scaffold solution or the control (DMEM or 800 μM hydrogen peroxide) for 24, 48, and 72 h. Following incubation, 10 µL PrestoBlue^TM^ solution was added to each well, and the plates were then placed back into the incubator for a further 30 min incubation. Fluorescence was measured using a microplate reader (Thermo Fisher Scientific, Loughborough, UK) at 560 nm excitation and 590 nm emission. Each formulation was tested in triplicate.

#### 2.5.2. Cell Migration

The HDF cells were seeded in 12-well plates at a density of 1×10^5^ cells per well and then incubated at 37 °C until the cells reached 100% confluence. Mitomycin-C (Sigma-Aldrich, Saint Louis, MO, USA) was added at a final concentration of 5 μg per mL, and the cells were incubated for an additional 4 h to inhibit cell proliferation. A sterile plastic 10 μL pipette tip was used to scratch the confluent cell monolayer evenly in each well, to generate a cell-free zone that was approximately 1 mm in width. The medium was aspirated and replaced with the released scaffold solution or the control (DMEM) in triplicate for 24 h. An inverted microscope (Nikon Corporation, Tokyo, Japan) was used to evaluate the distance of cell migration.

### 2.6. Statistical Analysis

SPSS version 22.0 (SPSS Co. Ltd., Bangkok, Thailand) was performed for all statistical evaluations. The statistical differences were calculated at *p* < 0.05. Results were analysed using one-way ANOVA.

## 3. Results

### 3.1. Analysis of Physical Properties

#### 3.1.1. Swelling Properties

**Figure 2:**
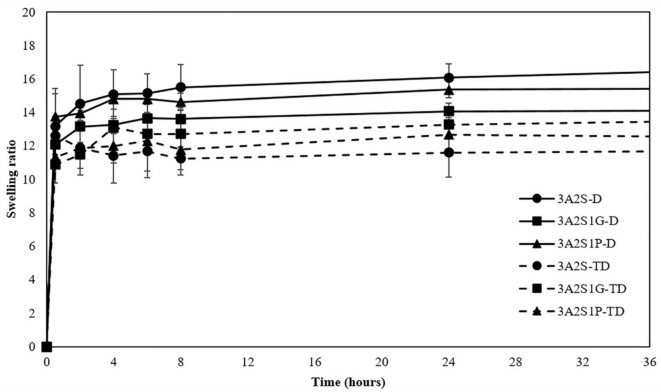
Swelling ratio of scaffolds (3A2S, 3A2S1G, 3A2S1P) that were prepared by the freeze-drying method (D) (solid line) and by freeze-thawing together with the freeze-drying method (TD) (dashed line).

Figure 2 shows the swelling ratio of all scaffolds. The time to reach the maximum swelling ratio of each scaffold was 24 h after starting. At 24 h, the swelling ratios of the 3A2S, 3A2S1G, and 3A2S1P of each method were not significantly different. However, comparing the D and TD methods, the swelling ratio of 3A2S-D was significantly higher than 3A2S-TD (16.08 ± 0.81 and 11.60 ± 1.45; *p* < 0.05). Moreover, a similar pattern was found in the swelling ratio of 3A2S1P-D compared to 3A2S1P-TD (15.37 ± 0.51 and 12.67 ± 0.99; *p* < 0.05).

#### 3.1.2. Protein Release

**Figure 3:**
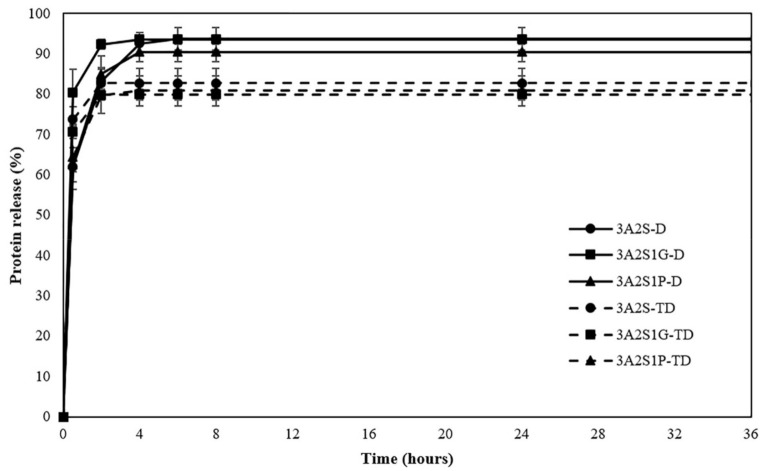
Protein release of scaffolds (3A2S, 3A2S1G, 3A2S1P) that were prepared by the freeze-drying method (D) (solid line) and by freeze-thawing together with the freeze-drying method (TD) (dashed line).

The protein release profiles of all scaffolds are shown in Figure 3. Comparing the three formulations (3A2S, 3A2S1G, 3A2S1P) obtained by both preparation methods, after 24 h, the percentage of protein release of all scaffolds were not significantly different. Nonetheless, a comparison between the D and TD methods showed that the protein release of 3A2S-D was significantly superior to 3A2S-TD (93.65 ± 2.83 and 82.72 ± 3.55; *p* < 0.05). Moreover, 3A2S1G-D and 3A2S1P-D released higher percentages of protein than 3A2S1G-TD and 3A2S1P-TD (93.51 ± 1.09, 90.46 ± 2.36, 79.87 ± 1.40 and 80.84 ± 3.71, respectively; *p* < 0.05). These results conformed to the swelling ratio results in that a lower swelling ratio tended to give a lower protein release.

#### 3.1.3. Gel Fraction Analysis

**Figure 4:**
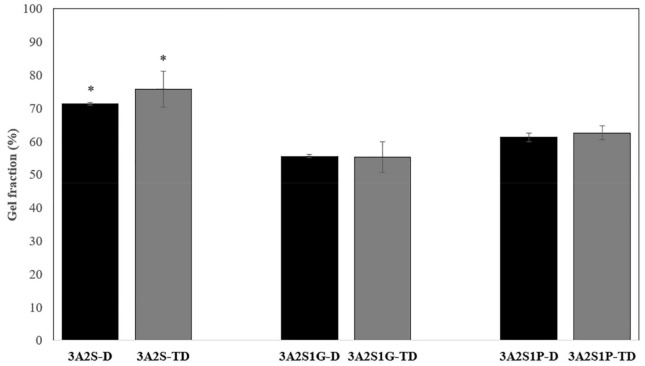
Percentage of gel fraction of scaffolds (3A2S, 3A2S1G, 3A2S1P) that were prepared by the freeze-drying method (D) (black box) and by freeze-thawing together with the freeze-drying method (TD) (grey box) (* significant difference compared to other formulations; *p* < 0.05).

Comparing the three formulations (3A2S, 3A2S1G, 3A2S1P) obtained by both preparation methods, the highest percentage of gel fraction was presented in the 3A2S scaffold (*p* < 0.05) (Figure 4). However, there was no statistically significant difference between gel fractions of the 3A2S1G and 3A2S1P scaffolds. Therefore, both glycerin and propylene glycol decreased the gel fraction of the scaffold. The gel fraction of scaffolds that were prepared using the TD method and the D method were not significantly different.

#### 3.1.4. Structural Analysis Using Fourier-Transform Infrared Spectroscopy

**Figure 5:**
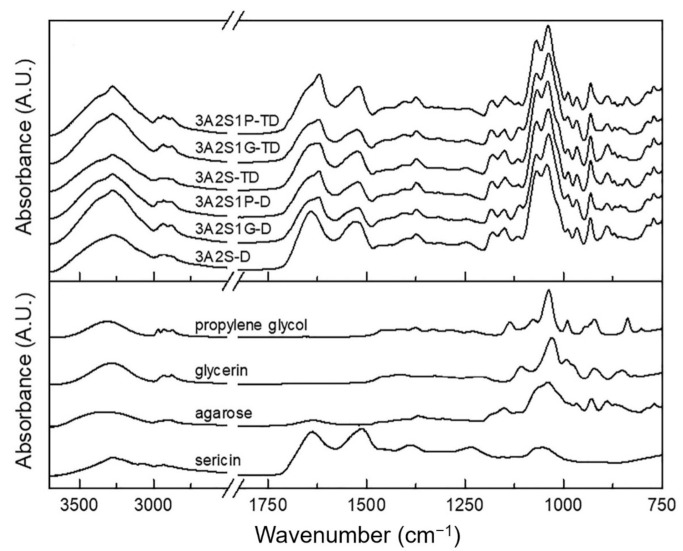
IR spectra of scaffolds (3A2S, 3A2S1G, 3A2S1P) that were prepared by the freeze-drying method (D) and by freeze-thawing together with the freeze-drying method (TD), and the raw materials used to prepare the scaffolds.

From Figure 5, it can be seen that all IR spectra of scaffolds show NH stretching, amide I, amide II, and amide III bands at 3276, 1641–1619, 1529–1519, and 1249–1246 cm^−1^, respectively, which are characteristics of protein from sericin. The peaks below 1200 cm^−1^ mainly came from agarose, glycerin, or propylene glycol, and their band assignments are listed in Supplement Materials Table S1.

**Figure 6:**
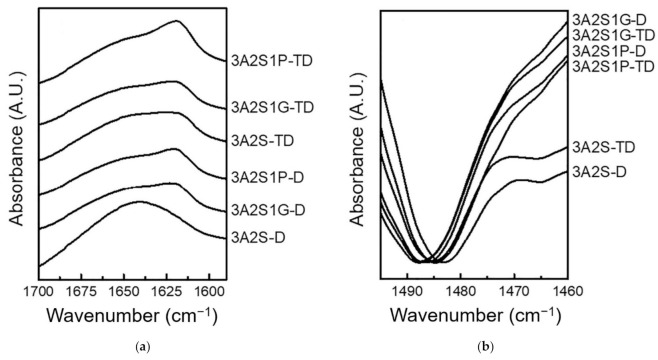
IR spectra of scaffolds (3A2S, 3A2S1G, 3A2S1P) that were prepared by the freeze-drying method (D) and by freeze-thawing together with the freeze-drying method (TD) in the regions of (**a**) 1700–1590 and (**b**) 1500–1460 cm^−1^.

Interestingly, the peak shifts of the NH stretching, amide II, and amide III bands suggests the formation of a hydrogen bond between the NH group of sericin and other compounds in each scaffold. From Figure 6a, by considering the amide I band, 3A2S-D showed the amide I peak at the highest wavenumber compared to the others, which indicates that the hydrogen bonds between the C = O of sericin and other compounds in this scaffold are weaker than other scaffolds, resulting in its highest swelling property. Moreover, scaffolds prepared by the TD method showed this peak at a lower wavenumber than those prepared by the D method in every formulation, suggesting that the hydrogen bonds between the C = O of sericin and other compounds in each scaffold prepared by the TD method are stronger than those prepared by the D method, leading to a lower swelling property.

By normalizing all IR spectra using peak heights of the amide I band, the intensities of shoulder at ~1470 cm^−1^ owing to the CH_2_ deformation vibration of agarose were compared, as shown in Figure 6b. In agarose with high crystallinity, the band at ~1470 cm^−1^ is intense, and vice versa. From the results, it was found that 3A2S1G and 3A2S1P showed significantly higher intensity than 3A2S for both the D and TD methods, indicating that the agarose in 3A2S1G and 3A2S1P has significantly higher crystallinity than in 3A2S. However, with the same formulation, there is no significant difference in crystallinity caused by the preparation method.

### 3.2. Analysis of Mechanical Properties

Table 1 shows the mechanical properties of all scaffolds, including tensile strength, elongation, and Young’s modulus. Comparing the three formulations that were prepared by the D method (3A2S-D, 3A2S1G-D, 3A2S1P-D), 3A2S1P-D significantly exhibited the highest tensile strength and Young’s modulus (*p* < 0.05), while 3A2S1G-D significantly showed the highest percentage of elongation. However, the tensile strength and Young’s modulus of the three formulations that were prepared by the TD method (3A2S-TD, 3A2S1G-TD, 3A2S1P-TD) were not significantly different. Nonetheless, the highest percentage of elongation was found in 3A2S1G-TD.

Comparing between the D or TD methods, the tensile strength and Young’s modulus of scaffolds prepared by the D method were higher than those prepared by the TD method. However, the percentage of elongation of scaffolds (same formulation) prepared by either the D method or the TD method were not significantly different.

### 3.3. Analysis of Biological Properties

#### 3.3.1. Cytotoxic Assay

**Figure 7:**
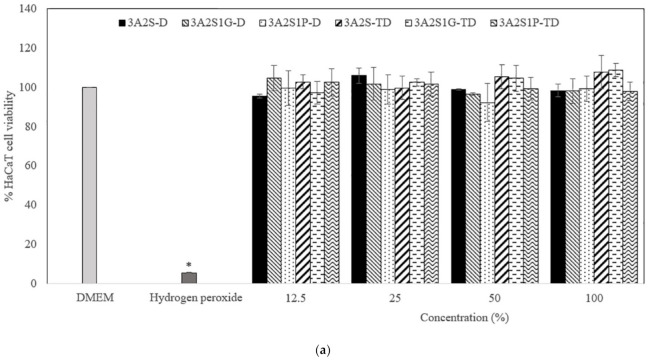

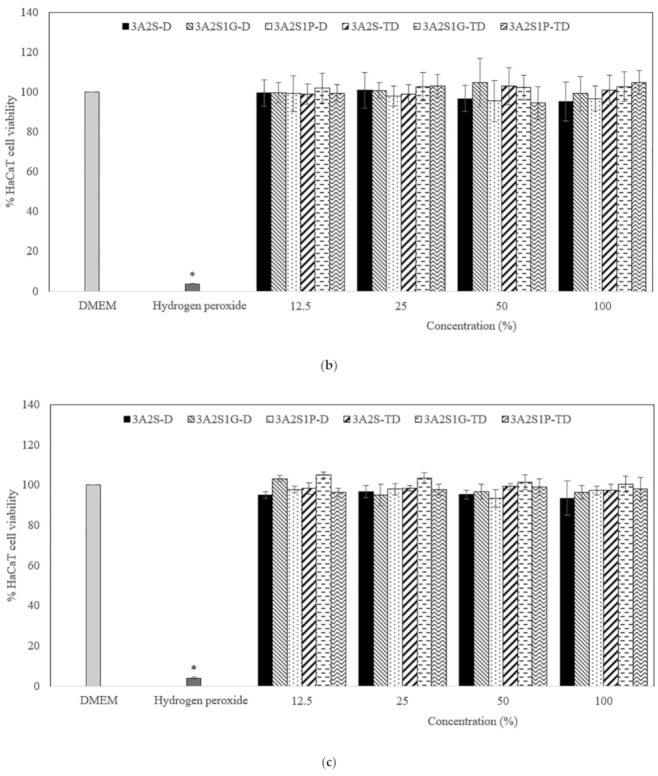
The percentage viability of HaCaT cells after treatment with different concentrations (12.5%, 25%, 50%, and 100%) of the released scaffolds (3A2S, 3A2S1G, 3A2S1P) that were prepared by the freeze-drying method (D) and by freeze-thawing together with the freeze-drying method (TD) at (**a**) 24 h, (**b**) 48 h, and (**c**) 72 h (* significant difference compared to other samples; *p* < 0.001).

**Figure 8:**
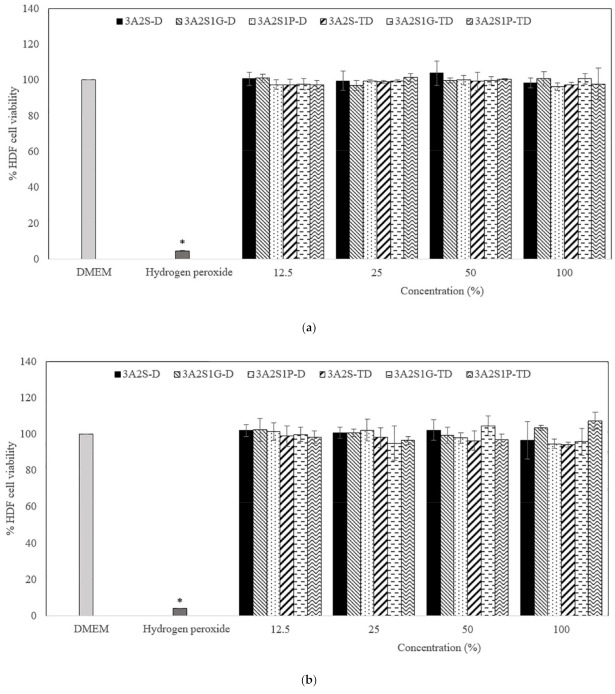

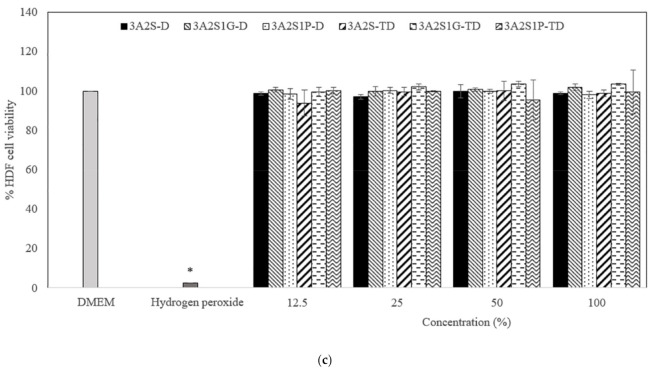
The percentage viability of HDF cells after treatment with different concentrations (12.5%, 25%, 50%, and 100%) of the released scaffolds (3A2S, 3A2S1G, 3A2S1P) that were prepared by the freeze-drying method (D) and by freeze-thawing together with the freeze-drying method (TD) at (**a**) 24 h, (**b**) 48 h, and (**c**) 72 h (* significant difference compared to other samples; *p* < 0.001).

Figure 7 and Figure 8 show the percentage viability of HaCaT and HDF cells after treatment with different concentrations (12.5%, 25%, 50%, and 100%) of the released scaffolds (3A2S-D, 3A2S1G-D, 3A2S1P-D, 3A2S-TD, 3A2S1G-TD, and 3A2S1P-TD). At 24, 48, and 72 h after treatment, all concentrations of the released scaffolds presented an HaCaT and HDF cell viability of more than 80%. The HaCaT and HDF cell viability of the scaffolds had no significant difference to the cell culture medium (DMEM). Moreover, they were always significantly higher than with hydrogen hydroxide (*p* < 0.05). There was no significant difference in the percentage viability of HaCaT and HDF cells after treatment with the released scaffolds when comparing between 24, 48, and 72 h. Therefore, all scaffolds were non-toxic to HaCaT and HDF cells.

#### 3.3.2. Cell Migration

**Figure 9:**
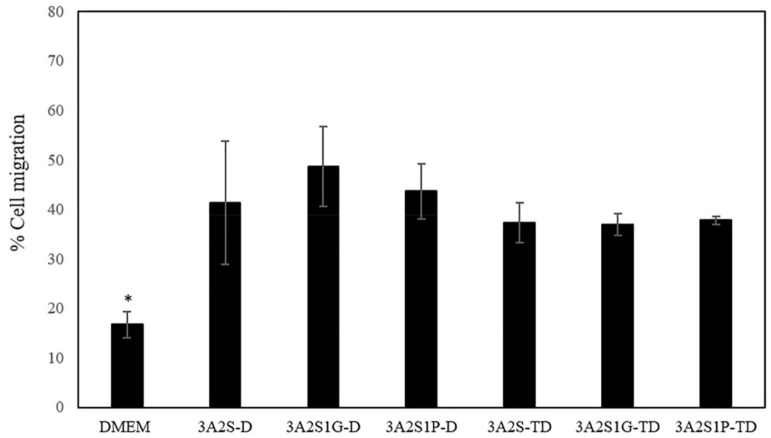
The percentage of cell migration after treatment with the released scaffolds (3A2S, 3A2S1G, 3A2S1P) that were prepared by the freeze-drying method (D) and by freeze-thawing together with the freeze-drying method (TD) at 24 h (* significant difference compared to other samples; *p* < 0.001).

An in vitro cell migration test is usually used as a model for wound healing evaluation. A higher percentage of cell migration is referred to as having superior wound healing promotion. All released scaffolds showed a significantly higher percentage of cell migration than DMEM (*p* < 0.05) (Figure 9). In addition, the percentage of cell migration of the scaffolds that were prepared by the D method (3A2S-D, 3A2S1G-D, 3A2S1P-D) tended to be superior to the scaffolds that were prepared by the TD method (3A2S-TD, 3A2S1G-TD, 3A2S1P-TD). These results might be the effect of their higher releasing profile. No significant difference was found when comparing the three formulations (3A2S-D, 3A2S1G-D, 3A2S1P-D). Accordingly, all scaffold formulations could accelerate wound healing. The scaffolds that were prepared by the TD method seemed to be slower at promoting wound healing than those formulations prepared by the D method. This property may be applied to develop further material as a controlled drug release scaffold.

## 4. Discussion

This study was successful in developing a natural wound dressing using an eco-friendly method. Physical crosslinking methods, the D method and the TD method, were compared. The swelling ratio of 3A2S-D and 3A2S1P-D were significantly higher than 3A2S-TD and 3A2S1P-TD (*p* < 0.05) (Figure 2). Moreover, the protein release of 3A2S-D, 3A2S1G-D, and 3A2S1P-D were significantly superior compared to 3A2S-TD, 3A2S1G-TD, and 3A2S1P-TD (Figure 3). The method involving freeze-thawing together with freeze-drying (TD) had an effect on the network structure of all scaffolds, resulting in stronger structures. These results were confirmed in Figure 6a where it is demonstrated that the hydrogen bonds between the C=O of sericin and other compounds in each scaffold prepared by the TD method are stronger than those prepared by the D method. For the freeze-drying method of agarose and proteins, Liu et al. [52] reported that gel networks of agarose were rapidly formed at the freezing phase, whereas silk proteins were dispersed and trapped in the agarose gel. The silk protein then created a network structure with ice crystals formed between and around the molecules, which would grow bigger at the maximum freezing state. At the drying phase, ice crystals directly create pores between the structures. Thus, the water adsorption property of agarose and sericin also affected the porosity [41]. Agarose scaffolds prepared by the freeze-drying method commonly show a high swelling profile [53]. The effects of the freeze-thawing method on the agarose structure were revealed by Yokoyama et al. [54]. They found that the agarose had a higher chain orientation at the freezing phase, resulting in the promotion of interfibrillar bonds, both within and between sheets; cryogels were then formed at the thawing phase. However, they indicated that agarose created the junction zones in the gel network before freezing, resulting in large pores and weak cohesion between agarose chains. An increase in freezing-thawing cycles increased the fibrillar cohesion, causing a stronger network structure and less swelling of the agarose gel. These results also agree with Neres Santos et al. [55] who reported that an increase in freeze-thawing cycles led to less porosity. That mechanism was also found in the forming of collagen and gelatin-based cryogel prepared by the freeze-thawing method [56]. The helical structure was the main structure of agarose gelation due to its hydrogen bonding and electrostatic interaction [57]. Therefore, the network structure of scaffolds prepared using the TD method in this study might have a higher fibrillar cohesion than the scaffolds prepared using only the D method. Accordingly, the swelling ratio and protein release of those scaffolds were lower than the swelling ratio and protein release of scaffolds prepared using only the D method. This property may be applied in the development of further material as a controlled drug release scaffold, such as controlling active agent release and cell delivery [56,58]. However, the tensile strength and Young’s modulus of scaffolds prepared by the TD method were lower than those prepared by the D method, without significant difference in elongation.

Comparing the three formulations (3A2S, 3A2S1G, 3A2S1P) of both preparation methods, the swelling ratio and protein release of all scaffolds were not significantly different (Figure 1 and Figure 2). Glycerin and propylene glycol are highly soluble in water, so they were lost during the swelling and releasing studies [59,60], resulting in no significant difference in swelling ratio and protein release compared to the non-plasticizer formulation. These results were also confirmed by Guadarrama-Acevedo et al. [61] who demonstrated that adding propylene glycol into polyvinylpyrrolidone did not affect the swelling ratio of the materials. The highest percentage of gel fraction was presented in the 3A2S scaffold, while there was no statistically significant difference between the gel fraction of 3A2S1G and 3A2S1P scaffolds (Figure 4). FTIR spectroscopy is more than just a tool for identifying chemical bonds and functional groups in materials. It can also be utilized to look into the chemical surroundings of the bonds of interest. IR bands can be shifted to higher or lower wavenumbers due to the surroundings of the interested bonds in the sample such as connecting to an electron-withdrawing/donating group and forming a hydrogen bond with another functional group. In this work, the degree of crystallinity of agarose in the scaffold was determined by observing the packing of cellulose chains from CH_2_ deformation vibration in crystalline cellulose, which is one of the cellulose IR bands used to determine cellulose crystallinity [62,63,64,65]. The high crystallinity of agarose, shown with the high IR intensity at ~1470 cm^−1^, might cause a decrease in intermolecular forces between agarose and other compounds. Accordingly, the other compounds would be easily disintegrated, causing a lower gel fraction. In addition, glycerin caused high elongation in both methods. The high elongation refers to the flexibility and stretch ability of the material [50]. The mechanical properties of scaffolds might be affected by many factors, including both intramolecular forces and crystallinity. The association between glycerol content and mechanical properties was presented by Paolicelli et al. [59]; that adding plasticizer improved material flexibility. Moreover, a higher concentration of glycerol was associated with more elasticity. Amin et al. [66] and Hidayati et al. [67] also presented the same results, showing that an increase in glycerol content had the effect of increasing elongation and decreasing the tensile strength of the materials because the plasticizer decreased the intermolecular strength and intermolecular interaction between molecules. The effects of propylene glycol on the mechanical properties conformed to the glycerol effect, where the percentage of material elongation significantly increased following an increase in propylene glycol concentration [61]. Comparing between glycerol and propylene glycol, Jantrawut et al. [50] reported that the tensile strength of films containing glycerol were less than those of films containing propylene glycol, while the elongation was higher than the elongation of films containing propylene glycol. Consequently, adding plasticizer had a positive effect on the mechanical properties while preserving most of the physical properties, resulting in a wound dressing advantage.

All scaffold formulations prepared by the D and TD methods were non-toxic to the cells because these scaffolds are composed of biocompatible material, prepared using physical crosslinking without toxic solvents. Moreover, both agarose and sericin are used in many biomaterial applications [42,68]. The wound healing property of sericin has been reported in many medical research studies [69,70,71]. Sericin increased the attachment of human skin fibroblasts, activated cell migration and proliferation, and activated collagen synthesis, resulting in wound healing acceleration without cytotoxicity [72,73,74]. In an in vivo study, sericin gel promoted a faster diabetic wound healing time than bacitracin zinc and polymyxin B [71]. The wound healing property of sericin in humans was also presented in a study by Siritientong et al. [75]. They showed that a split-thickness skin graft donor site wound treated with a wound dressing containing sericin, exhibited a better wound healing time than a dressing without sericin. The results of this study also found that the cell migration of all scaffolds containing sericin was significantly higher than the control (DMEM), resulting in wound healing acceleration. Moreover, the TD scaffolds may be applied to develop further material as a controlled drug release scaffold that may control an acceleration of cell migration, leading to the design of a new and effective wound healing time pattern.

An eco-friendly product produced by green technology will reduce pollution and waste [18]. Finding a simple and appropriate eco-friendly method for manufacturing still has its challenges. This study presents the successful production of natural scaffolds using D and TD eco-friendly methods that were effective for wound dressing production. Properties of the developed dressings in this study were close to that of the ideal hydrogel wound dressing shown in Table 2 [76,77,78]. Their properties were non-toxicity, biocompatibility, elasticity, and being friendly to the environment. Moreover, the developed dressings could deliver active agents and could promote wound healing. However, the limitation of this study is that a low number of samples were analysed because this study was processed in a laboratory. Further exploration is required for large-scale application, including manufacture. Furthermore, other properties, such as product stability, oxygen permeability, skin attachment, and an in vivo study of efficacy and safety will be investigated in a future study.

## 5. Conclusions

The successful processing of natural scaffolds using environmentally friendly production was demonstrated in this study. Both D and TD methods could be used as the production of natural scaffolds. The scaffolds produced by the TD method had stronger bonding between sericin and other compounds, resulting in a low swelling ratio and low protein release of the scaffolds. Agarose and sericin are natural agents that could be developed as biomaterial scaffolds. Adding plasticizers, especially glycerin, into the scaffolds had the effect of decreasing the gel fraction of the scaffolds due to an increase in agarose crystallinity. Moreover, the plasticizer increased elongation properties, leading to an improvement in mechanical properties, including an increase in softness and skin attachment of the scaffold. All scaffolds were non-cytotoxic and could activate cell migration, referring to wound healing properties. These properties may be applied to develop further material as a controlled drug release scaffold.

## Figures and Tables

**Table 1:** Mechanical properties of all scaffolds.

Scaffolds	Tensile Strength (MPa)	Elongation (%)	Young’s Modulus (N/mm^2^)
3A2S-D	1.54 ± 0.55 ^#^	3.53 ± 1.19	54.68 ± 11.17 *^, #^
3A2S1G-D	1.35 ± 0.14	6.76 ± 0.96 *	29.17 ± 10.44 *
3A2S1P-D	2.77 ± 0.94 *^, #^	4.11 ± 0.86	81.43 ± 20.82 *^, #^
3A2S-TD	0.51 ± 0.18	2.05 ± 0.53 *	26.92 ± 10.20
3A2S1G-TD	0.64 ± 0.13	6.13 ± 0.40 *	13.81 ± 2.62
3A2S1P-TD	0.71 ± 0.28	3.69 ± 0.56 *	24.16 ± 6.37

* significant difference comparing to other formulation (same method); *p* < 0.05, ^#^ significant difference comparing between D method and TD method (same formulation); *p* < 0.05.

**Table 2:** Comparing properties of the dressing in this study to the ideal hydrogel wound dressing [76,77,78].

Properties	Ideal Wound Dressing	Dressing in This Study
No toxic component	Yes	Yes
Eco-friendly product	Yes	Yes
Lack of cytotoxicity	Yes	Yes
Wound healing acceleration	Yes	Yes
Wound protection	Yes	Yes
Soft elasticity	Yes	Yes
Drug delivery	Yes	Yes
Excess exudate absorption	Yes	Yes
Moist healing environment	Yes	Yes
Pain reduction	Yes	Probable
Oxygen permeability	Yes	Probable
Easy removal	Yes	Probable

## Data Availability

The data presented in this study are available on request from the corresponding author.

## Supplementary Materials

The following are available online at https://www.mdpi.com/article/10.3390/polym13193370/s1, Table S1: Band assignments for IR spectra of agarose, glycerin, and propylene glycol [79].
