# Supplementary material for: Superior Technique for the Production of Agarose Dressing Containing Sericin and Its Wound Healing Property
Source: Polymers (Basel). 2021 Sep 30;13(19):3370. doi: 10.3390/polym13193370 (PMC8512865; doi:10.3390/polym13193370)
Supplement: Supplementary file 1 [file polymers-13-03370-s001.zip › polymers-1377227-supplementary.pdf]

Table S1. Band assignments for IR spectra of Sagarose, glycerin, and propylene glycol [79].

Wavenumber (cm^{-1})			Band assignment
Agarose	Glycerin	Propylene glycol	
3340	3282	3314	O–H str.
		2970	asym. CH_3 str.
2955	2931	2930	asym. CH_2 str.
2899	2878	2876	sym. CH_2 str.; sym. CH_3 str.
1636			O–H def. vib.
1465			CH_2 def. vib. (intensity affected by degree of crystallinity)
		1457	asym. CH_3 def. vib.
1413	1413	1411	CH_2 def. vib. (primary alcohols)
1368		1374	sym. CH_3 def.; CH def. vib. (secondary alcohols)
	1327	1332	CH_2 wagging vib. (primary alcohols); CH def. vib. (secondary alcohols)
1252		1287, 1232	CH def. vib. (secondary alcohols)
1151	1108	1136, 1077	C–O str. (secondary alcohols)
1039	1030	1039	CCO str. (primary alcohols)
966	992	989	CH_2 twisting vib. (primary alcohols)
		921	CH_3 rocking vib.
887	850	839	CCO str. (primary & secondary alcohols)
		803	CCO str.
771			ring vib.
